# Clinical significance on switching CDK4/6 inhibitors among 13,284 patients with metastatic breast cancer

**DOI:** 10.1007/s12282-025-01768-6

**Published:** 2025-10-12

**Authors:** Takuya Nishina, Maki Tanioka, Kenji Takada, Takahiro Tsukioki, Yuko Takahashi, Tadahiko Shien, Shinichi Toyooka

**Affiliations:** 1 Department of General Thoracic Surgery and Breast and Endocrinological Surgery, Graduate School of Medicine, Dentistry, and Pharmaceutical Sciences, Okayama University, 2-5-1 Shikata-Cho, Kita-Ku, Okayama, 700-8558 Japan; 2 Medical AI Project, Graduate School of Medicine, Dentistry, and Pharmaceutical Sciences, Okayama University, Okayama, 700-8558 Japan

**Keywords:** Cyclin-dependent kinase 4/6 inhibitors, Endocrine therapy, HR-positive/HER2-negative advanced breast cancer, Progression-free survival, Time to discontinuation

## Abstract

Recent clinical trials have shown that switching to a combination therapy of a cyclin-dependent kinase 4/6 inhibitor (CDK4/6i) and endocrine therapy (ET) prolongs progression-free survival (PFS) compared with ET monotherapy. Reports indicate that abemaciclib provides benefits regardless of the *PIK3CA* mutation status; however, its clinical benefits remain insufficient. This study aimed to evaluate the clinical significance of switching CDK4/6i + ET in a large real-world cohort. Using a medical database, we identified 13,284 patients with hormone receptor–positive/human epidermal growth factor receptor 2–negative advanced breast cancer who received CDK4/6i + ET between 2008 and 2022. Patients were categorized into five groups based on their first- and second-line therapy patterns. We compared the median time to discontinuation (TTD) among the groups. In patients who switched from one CDK4/6i + ET to another CDK4/6i + ET, the second-line TTD and total TTD of first- and second-line therapies (n = 542) were significantly longer than those in patients who switched from CDK4/6i + ET to ET monotherapy (n = 490) (the second-line TTD: 11.2 vs. 4.9 months, p < 0.01; total TTD: 25.1 vs. 20.5 months, p < 0.01). The order of palbociclib and abemaciclib administration did not significantly affect the second-line or total TTD in patients who switched from one CDK4/6i + ET to another CDK4/6i + ET. Switching from one CDK4/6i + ET to another CDK4/6i + ET resulted in a significantly longer TTD than switching to ET monotherapy. Considering the phase III clinical trial results of capivasertib, switching to CDK4/6i + ET is a viable therapeutic option regardless of the *PIK3CA* mutation status.

**Supplementary Information:**

The online version contains supplementary material available at 10.1007/s12282-025-01768-6.

## Introduction

According to the Hortobagyi algorithm, treatment for hormone receptor (HR)–positive, human epidermal growth factor receptor 2 (HER2)–negative advanced breast cancer (ABC) involves the selection of endocrine therapy (ET) in the absence of severe organ dysfunction or rapid disease progression (visceral crisis), whereas chemotherapy (CT) is chosen in cases of visceral crisis [1]. Cyclin-dependent kinase 4/6 inhibitors (CDK4/6is) regulate the cell cycle by inducing cell cycle arrest in the G1 phase [2]. The three available CDK4/6is are palbociclib, abemaciclib, and ribociclib. CDK4/6is have been shown to improve progression-free survival (PFS) in patients with HR-positive/HER2-negative ABC when used in combination with ET as a first- or second-line therapy [3–9]. Combination therapy using a CDK4/6i and ET is the standard of care for HR-positive/HER2-negative ABC [1]. However, the development of resistance to hormone therapy is a major challenge in the treatment of HR-positive/HER2-negative ABC. According to the results from various clinical trials, ET monotherapy in the second-line setting after first-line CDK4/6i + ET has a PFS of only 2.8–4.6 months [10–15]. Additionally, *PIK3CA/AKT1/PTEN* mutations have been recognized as one of the causes of resistance to hormone therapy [16]. In patients with HR-positive/HER2-negative ABC with *PIK3CA/AKT1/PTEN* mutations, the SOLAR-1 trial reported that alpelisib (PI-3 kinase inhibitor) significantly prolonged PFS (11.0 months vs. 5.7 months; hazard ratio [HR], 0.65; p < 0.001), the INAVO120 trial reported that inavolisib (PI-3 kinase inhibitor) significantly prolonged PFS (15.0 months vs. 7.3 months; HR, 0.43; p < 0.001), and the CAPItello-291 trial reported that capivasertib the (AKT inhibitor) significantly prolonged PFS (7.3 months vs. 3.1 months; HR, 0.50; p < 0.001) [10, 28, 31]. However, in all cases, the absolute improvement in PFS was modest, and the clinical significance remained limited. Given these suboptimal treatment options, currently, physicians choose to switch CDK4/6is (from one CDK4/6i + ET to another CDK4/6i + ET), extend CDK4/6is with a different ET (from one CDK4/6i + ET to the same CDK4/6i + another ET), or transition to ET monotherapy, CT, or PI3K/AKT inhibitors depending on the patient’s condition. In recent years, clinical trials have been conducted on switching or extending CDK4/6i + ET. Extension of CDK4/6i therapy has been investigated in the PACE and PALMIRA trials. In these trials, extending palbociclib from first- to second-line therapy while changing the ET combination failed to prolong PFS in second-line therapy [11, 12]. Switching to a CDK4/6i was investigated in the MAINTAIN, post-MONARCH, and EMBER-3 trials. In the MAINTAIN trial, switching from CDK4/6i + ET (palbociclib: 84%, ribociclib: 16%) to ribociclib + ET significantly prolonged the second-line PFS compared with changing to ET monotherapy [13]. In the post-MONARCH trial, switching from palbociclib + aromatase inhibitor (AI) or from ribociclib + AI to abemaciclib + fulvestrant (FUL) significantly prolonged the second-line PFS compared with switching to FUL monotherapy [14]. In the EMBER-3 trial, among patients accounting for 65% of the cohort who had received prior treatment with a CDK4/6i + AI, switching to abemaciclib + imlunestrant significantly prolonged PFS in the second-line setting compared with switching to imlunestrant monotherapy. [15]. However, these studies included a small number of patients. To overcome this, this study utilized a large real-world clinical database comprising 13,284 patients to evaluate the significance of switching to another CDK4/6i after the use of a CDK4/6i in the first-line therapy for HR-positive/HER2 − negative ABC.

## Methods

### Data source

This study utilized observational data from the Medical Data Vision Co., Ltd. (MDV) database. The MDV database is a de-identified database comprising discharge summaries and medical insurance billing data obtained from inpatient and outpatient visits at Japanese hospitals using the Diagnosis Procedure Combination (DPC) system [17]. The DPC system includes hospitals providing acute care and other services, incorporating many of Japan’s major medical and cancer centers. As of January 2021, the MDV database includes the data of approximately 34.84 million individuals [18]. This study was conducted in accordance with the guidelines of the Declaration of Helsinki and Good Pharmacoepidemiology Practices. According to Japan’s Ethical Guidelines for Medical and Health Research Involving Human Subjects [19], this noninterventional retrospective study using anonymized patient data did not require informed consent.

### Study design

This study is a retrospective cohort study. The clinical variables that can be extracted from the MDV database include age, sex, prescribed medications, dosage, prescription date, diagnosis, surgical procedure, and date of surgery. However, information on tumor size, lymph node involvement, disease stage, metastatic sites, date of recurrence, number of metastatic organs, and adverse events are not available. In this study, HR-positive/HER2-negative breast cancer was operationally defined as cases in which ET was administered and anti-HER2 therapies were not administered. This definition was adopted because the MDV database does not directly capture HR/HER2 status, and treatment patterns were used as a surrogate. Surgical data were extracted from the MDV database for patients with malignant breast tumors who underwent the following procedures: mastectomy, quadrantectomy, lumpectomy, and nipple-sparing mastectomy.

### Patients

The study population and their clinical information were extracted from the MDV database as follows.

(1) To accurately determine the therapy lines of patients, it was necessary to observe data prior to the launch of abemaciclib and palbociclib; therefore, the follow-up period was set from April 2008 to December 2022, which was the maximum period for which the data were available. (2) Prescription records of palbociclib or abemaciclib as well as those of ET (FUL, letrozole [LET], anastrozole [ANA], exemestane [EXE], tamoxifen [TAM], and toremifene [TOR]) were confirmed. Ribociclib has not been approved in Japan. Therefore, this study focuses on evaluating treatment outcomes related to palbociclib and abemaciclib. (3) The absence of prescriptions for anti-HER2 therapies (trastuzumab and pertuzumab) was confirmed. (4) The age at the time of CDK4/6i prescription was ≥ 20 years. (5) For patients prescribed CDK4/6is, information on CT (paclitaxel [PTX], docetaxel [DTX], gemcitabine [GEM], carboplatin [CBDCA], bevacizumab + paclitaxel [Bev + PTX], doxorubicin + cyclophosphamide [AC], epirubicin + cyclophosphamide [EC], eribulin [Eri], vinorelbine [VNR], capecitabine [Cape], and tegafur/gimeracil/oteracil [TS1]) administered during the follow-up period was also investigated.

### *Postoperative recurrence or *de novo* stage IV*

The MDV database does not provide the date on which distant metastasis was diagnosed. Therefore, we defined de novo stage IV and postoperative recurrence based on the use of denosumab and the date of surgery as follows; Postoperative recurrence was defined as patients who underwent surgery at the earliest point in their treatment course, excluding those who received neoadjuvant chemotherapy (NAC). NAC was defined as cases in which both taxane- and anthracycline-based chemotherapies were prescribed as the initial treatments in the clinical course, and surgery was performed within six months from the start date of these agents. De novo stage IV was defined as: (1) patients with no history of surgery, (2) patients with a history of surgery in whom denosumab was initiated prior to the surgery date (suggesting bone metastasis), (3) patients whose treatment was initiated prior to the surgery date (excluding NAC). Surgical procedures in de novo stage IV cases were considered to be for local control purposes.

### Adjuvant therapy and diagnosis of recurrence

Adjuvant endocrine therapy was defined as the prescription of LET, ANA, EXE, or TAM within three months after surgery. Adjuvant chemotherapy was defined as the prescription of taxane- or anthracycline-based chemotherapy within three months after surgery in patients with postoperative recurrence who had not received NAC. The date of diagnosis of postoperative recurrence was defined as the earliest prescription date of CT (excluding adjuvant chemotherapy), CDK4/6i, FUL, TOR, or denosumab during the postoperative treatment course. In December 2021, abemaciclib was approved in Japan as an adjuvant therapy for patients with high-risk, HR-positive and HER2-negative early breast cancer. Therefore, among patients who were prescribed abemaciclib between December 2021 and December 2022, those who were prescribed abemaciclib within 3 months after surgery or adjuvant chemotherapy were considered to have received it as adjuvant therapy.

### Endpoints

The primary endpoint was to compare the time to discontinuation (TTD) of second-line therapy between two groups of patients with HR-positive/HER2-negative ABC diagnosed with de novo stage IV disease or recurrence: those who received CDK4/6i + ET as first-line therapy and switched to another CDK4/6i + ET in the second-line therapy and those who switched to ET monotherapy. The secondary endpoint was to investigate the therapy patterns, details of ET combined with a CDK4/6i, and the total TTD from the start of first-line therapy to the end of second-line therapy.

### TTD

The TTD of the combination therapy using a CDK4/6i and ET was defined as the period from the start date of either the CDK4/6i or the ET combined with the CDK4/6i, whichever came first, to the end date when both the CDK4/6i and the ET were discontinued. The total TTD of the first- and second-line therapies was defined as the period from the start of first-line therapy to the end of second-line therapy. A limitation is that the DPC system records only the first and last prescription dates. Therefore, there were no data regarding the exact date of drug discontinuation or the reasons for discontinuation. Accordingly, the date of the first prescription of a drug was defined as the start date of drug administration, and the date of the last prescription was defined as the end date of drug administration.

### Excluded patients

Patients with overlapping prescriptions for three or more drugs during the same period were excluded owing to data inaccuracies. Patients with an unexplained gap of 60 days or more between the end of first-line therapy and the start of second-line therapy were excluded. Patients with an overlap of 7 days or more between the end of first-line therapy and the start of second-line therapy were excluded. Patients with unknown ET combined with CDK4/6i are excluded. Abemaciclib was approved in Japan in November 2018, and palbociclib was approved in September 2017. Patients who had been using ET before October 2018 and those who received abemaciclib were excluded, as this did not provide accurate data for the combination therapy of CDK4/6i + ET. Similarly, patients who had been using ET before August 2017 and then received palbociclib were excluded. Patients who received CDK4/6i + ET as the first-line therapy without any changes in the regimen were excluded.

### Statistical analysis

Patients with a final prescription date of December 2022 were considered to have ongoing drug administration and were considered as censored cases. HRs and 95% confidence intervals (CIs) between the two groups were examined using a Cox proportional hazards model for regression analysis. The Kaplan–Meier method was used to estimate the median and 95% CIs for the TTD. Differences between the groups were evaluated using the log-rank test. No adjustments were made for biases or confounding factors, and missing data were not Imputed. Data were processed using SQLite 3.33.0 and Python 3.12.2.

## Results

### Patient selection

**Fig. 1:**
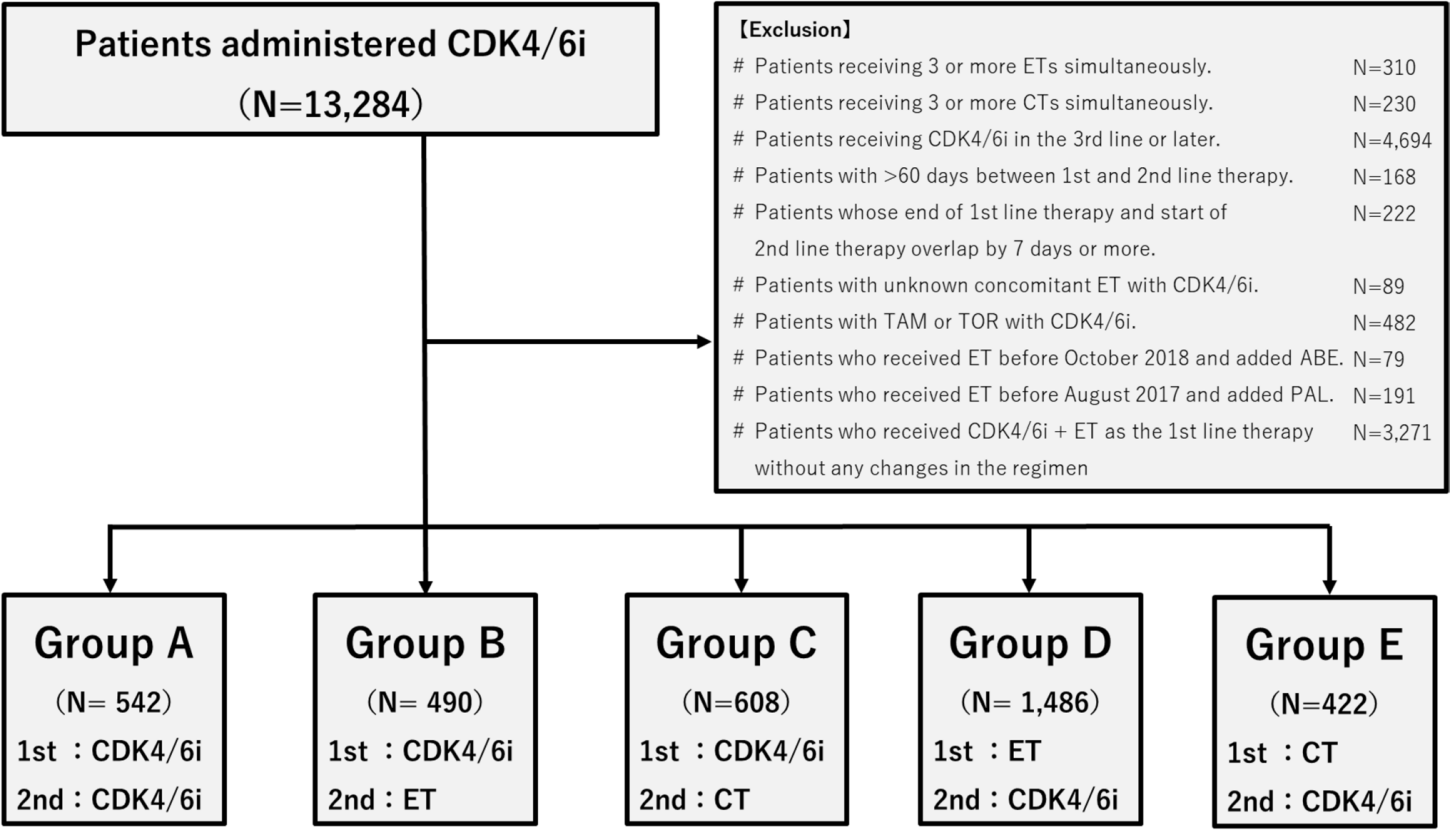
This Consort diagram presents patient selection. A total of 13,284 patients, aged 20 years or older, received combination therapy with CDK4/6i and ET between April 2008 and December 2022, without receiving anti-HER2 therapy. After excluding ineligible cases, 13,284 patients were classified into six groups based on their therapy patterns from first-line to second-line therapy. Patients with a history of abemaciclib administration only or palbociclib administration only were classified into groups B, C, D, or E. Most patients who received both CDK4/6i drugs were classified into group A. However, 27 patients who received CDK4/6i in the first-line setting, switched to ET in the second-line setting, and then received another CDK4/6i from the third line onward were classified into group B. 32 patients who received CDK4/6i in the first-line setting, switched to CT in the second-line setting, and then received another CDK4/6i from the third line onward were classified into group C. 249 patients who received ET in the first-line setting, received their first CDK4/6i in the second-line setting, and then received another CDK4/6i from the third line onward were classified into group D. 56 patients who received CT in the first-line setting, received their first CDK4/6i in the second-line setting, and then received another CDK4/6i from the third line onward were classified into group E

Figure 1 illustrates the recruitment process of the study cohort. A total of 13,284 patients with a history of CDK4/6i administration were identified in the database. Among these, 5,153 patients had a history of abemaciclib administration only, 6,126 had a history of palbociclib administration only, and 2,005 patients had a history of both abemaciclib and palbociclib administration. Thus, the 13,284 patients were classified into the following five groups:

Group A: patients who switched from one CDK4/6i + ET to another CDK4/6i + ET. (N = 542).

Group B: patients who switched from CDK4/6i + ET to ET monotherapy. (N = 490).

Group C: patients who switched from CDK4/6i + ET to CT. (N = 608).

Group D: patients who switched from ET monotherapy to CDK4/6i + ET. (N = 1,486).

Group E: patients who switched from CT to CDK4/6i + ET. (422).

### Characteristics of patients

Patient characteristics are summarized in Table 1. The median age of all patients was 64 years (range, 21–100 years), and most of the patients were female (99.5%). Groups A, B, and C had a history of CDK4/6i use in the first-line setting. Among these groups, ET combined with a CDK4/6i as the first-line therapy was evenly split between FUL (50.0%) and AI (50.0%). Groups A, D, and E had a history of CDK4/6i use in the second-line setting. In these groups, FUL was the predominant ET combined with a CDK4/6i in the second-line therapy (FUL, 66.2%; AI, 33.8%). In all groups, patients with de novo stage IV disease outnumbered those with postoperative recurrence.

**Table 1:**
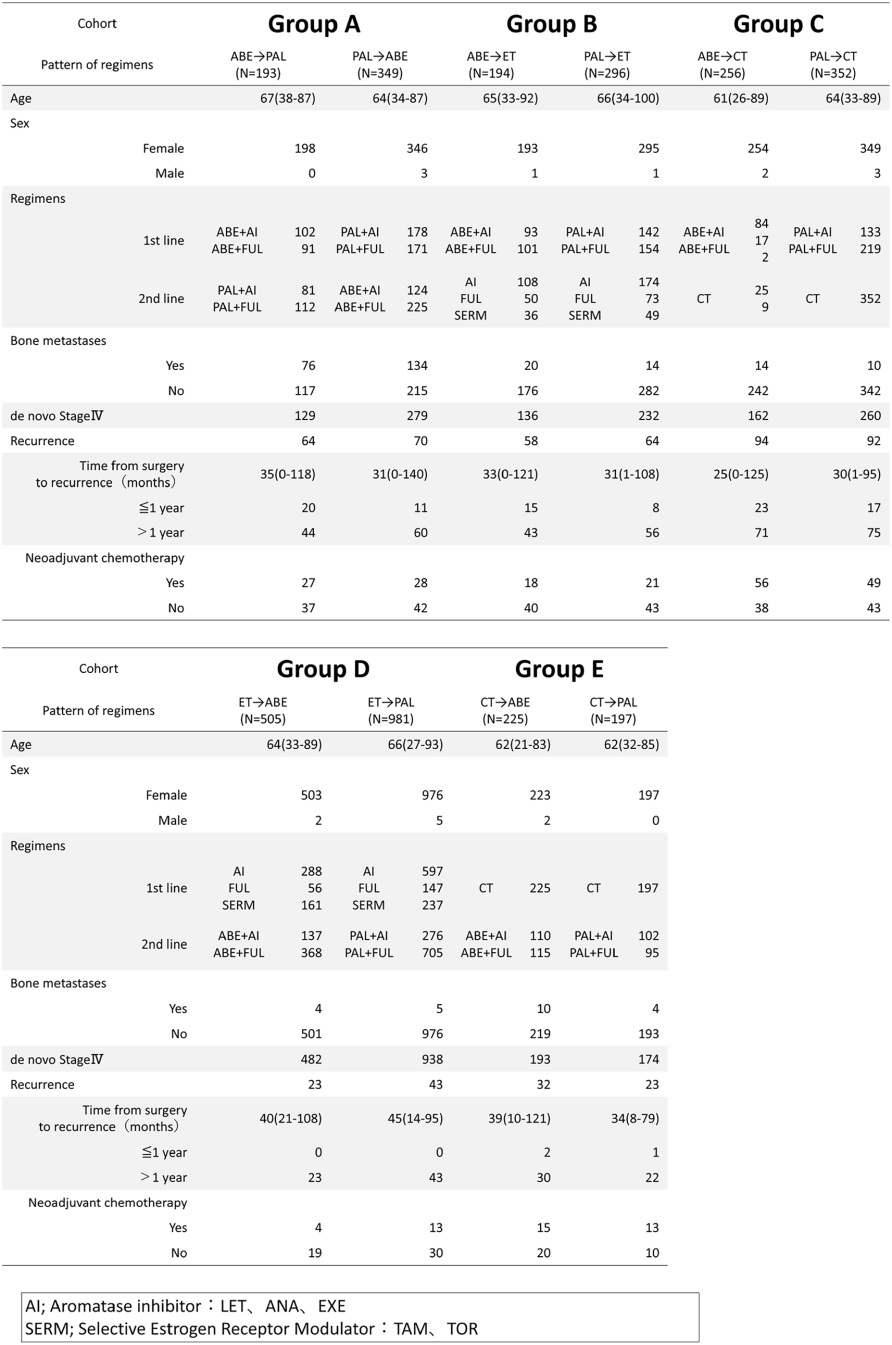
Characteristics of patients

### Regimen patterns

**Fig. 2:**
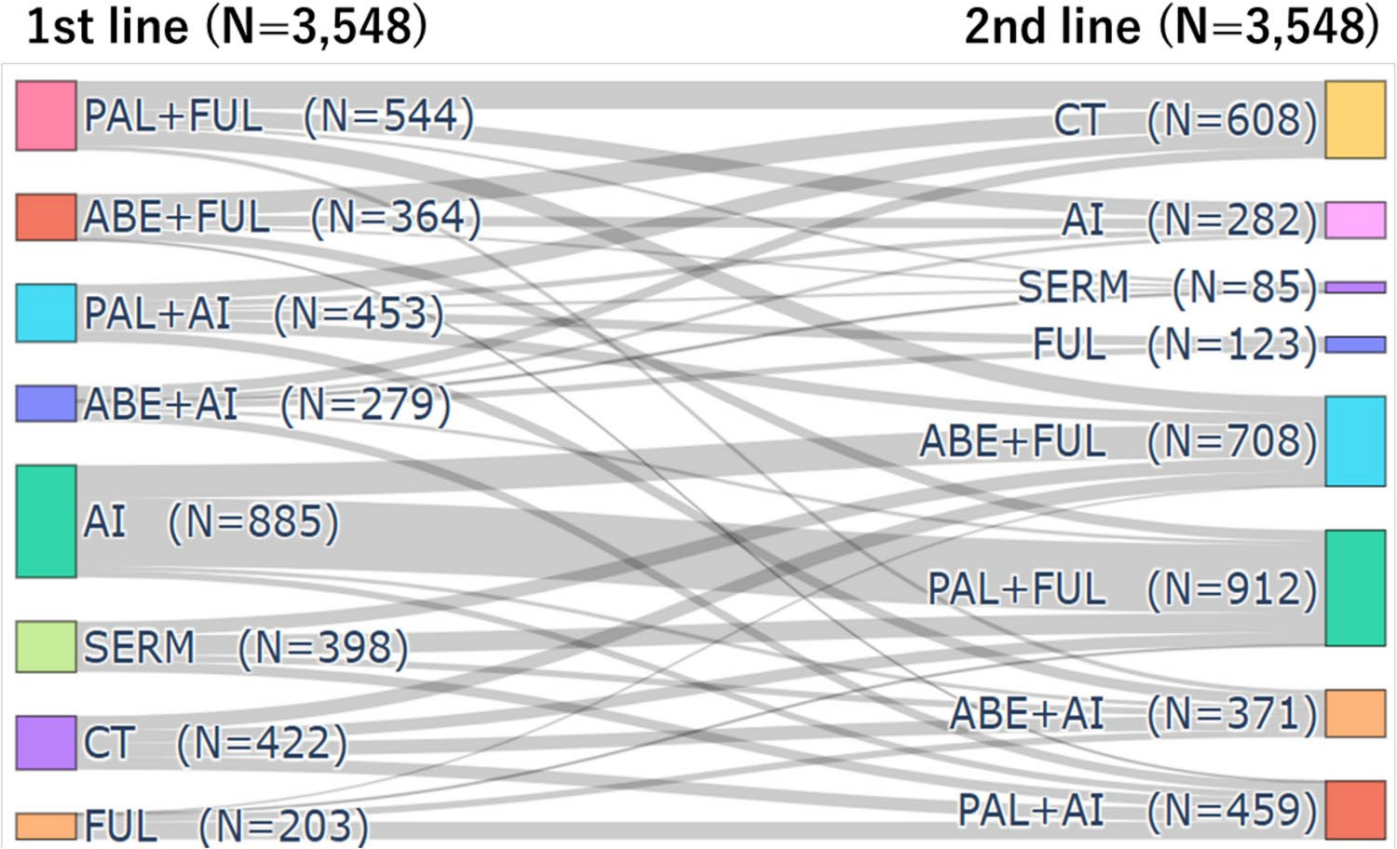
This Sankey diagram presents the patterns of regimens from first-line to second-line therapy. The left side of the diagram shows the first-line regimens, while the right side displays the second-line regimens, along with the number of patients for each regimen. CDK4/6is are distinguished as abemaciclib (ABE) and palbociclib (PAL). Chemotherapy (CT) details include paclitaxel (PTX), bevacizumab + paclitaxel (Bev + PTX), doxorubicin + cyclophosphamide (AC), epirubicin + cyclophosphamide (EC), eribulin (Eri), vinorelbine (VNR), capecitabine (Cape), and tegafur/gimeracil/oteracil (TS1). aromatase inhibitors (AI) include letrozole (LET), anastrozole (ANA), and exemestane (EXE). Selective Estrogen Receptor Modulators (SERM) include tamoxifen (TAM) and toremifene (TOR). Fulvestrant (FUL) is classified as a selective estrogen receptor degrader (SERD)

Figure 2 shows the patterns of regimens from first-line to second-line therapy for all patients. A total of 1,640 patients were prescribed a CDK4/6i in the first-line setting. A total of 1,908 patients were prescribed a CDK4/6i for the first time in the second-line setting. The majority (46.4%) of patients received AI as first-line therapy (Table S1). Among patients who switched to another CDK4/6i, 35.8% switched by changing the combined ET, whereas 64.2% switched without changing the combined ET. In the first-line setting, FUL was more commonly combined with a CDK4/6i than AI (AI, 42.5%; FUL, 57.5%). In the second-line setting, FUL remained to be the more commonly combined drug with a CDK4/6i than AI (AI, 25.9%; FUL, 74.1%) (Fig. S1).

### Analysis of TTD

**Fig. 3:**
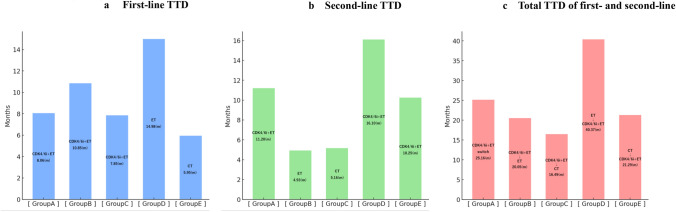
The therapy regimens and median TTD values for each group are presented as bar graphs. **a** shows the first-line TTD, **b** shows the second-line TTD, and **c** shows the total TTD of first- and second-line therapies (the total TTD). Since group F consists of patients who received only CDK4/6i + ET therapy during the observation period, they do not have a second-line TTD. Therefore, the first-line TTD and the total TTD for group F are the same. Furthermore, the total TTD is defined as the period from the start of first-line therapy to the end of second-line therapy. As a result, the sum of the first-line TTD and the second-line TTD does not necessarily match the total TTD. This is due to the use of the median in the TTD analysis. We compared the TTD between groups A and B, groups A and C, groups B and D, and groups A and E. For groups B and D and groups A and E, only the total TTD were compared owing to differences in the therapy line where a CDK4/6i was used

**Fig. 4:**
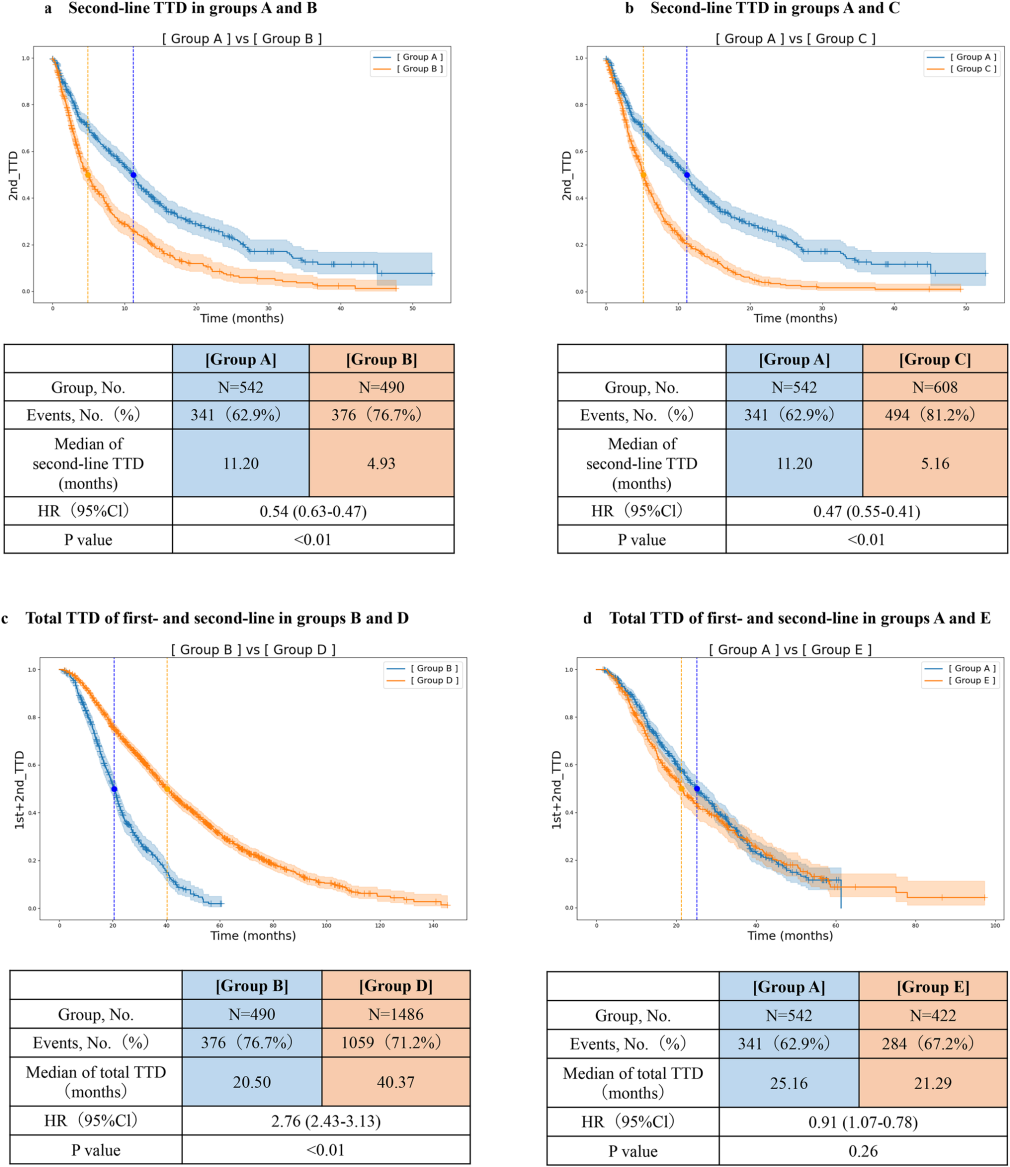
Kaplan–Meier estimates of Time to Discontinuation (TTD) are shown in **a** for groups A and B (second-line TTD), **b** for groups A and C (second-line TTD), Fig. 4c for groups B and D (total TTD of first- and second-line), and Fig.  4 d for groups A and E (total TTD of first- and second-line)

The TTD for each group was determined using the Kaplan–Meier method and is presented as a bar graph (Fig. 3). The median TTD of groups A and B was compared. Group A showed a significantly longer second-line TTD of 11.20 months compared with group B at 4.93 months (HR, 0.54; 95% CI, 0.47–0.63; p < 0.01) (Fig. 4a). Group A showed a significantly longer the total TTD of first- and second-line therapies (total TTD) of 25.16 months compared with group B at 20.50 months (HR, 0.72; 95% CI, 0.62–0.83; p < 0.01) (Fig. S2). The Impact of the order of palbociclib and abemaciclib administration on the TTD was examined in group A. There was no significant difference in the second-line TTD between patients who switched from palbociclib to abemaciclib at 11.00 months and those who switched from abemaciclib to palbociclib at 11.33 months (HR, 1.04; 95% CI, 0.83–1.32; p = 0.67) (Fig. S3a). There was no significant difference in the total TTD between patients who switched from palbociclib to abemaciclib at 25.66 months and those who switched from abemaciclib to palbociclib at 23.10 months (HR, 0.89; 95% CI, 0.71–1.32; p = 0.34) (Fig. S3b). In group A, statistical significance was tested for the median TTD across variables such as age (< 65 years vs. ≥ 65 years), path of onset (recurrence vs. de novo stage IV disease), presence of bone metastasis, history of NAC among patients with recurrence, and time from the first surgery to recurrence (≥ 1 year vs. < 1 year). Although not statistically significant in the multivariate analysis, patients younger than 65 years, those with postoperative recurrence, those with bone metastasis, and those with recurrence without NAC showed a longer TTD. Patients who showed recurrence within 1 year after surgery had a significantly higher total TTD than those who showed recurrence after 1 year (HR, 1.99; 95% CI, 1.13–3.52; p = 0.01) (Table 2).

**Table 2:**
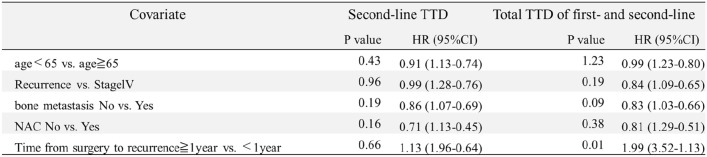
Characteristics of patients

The median TTD of groups A and C was compared. Group A showed a significantly longer second-line TTD of 11.20 months than group C at 5.16 months (HR, 0.47; 95% CI, 0.41–0.55; p < 0.01) (Fig. 4b). Similarly, group A showed a significantly longer total TTD of 25.16 months than group C at 16.49 months (HR, 0.58; 95% CI, 0.51–0.67; p < 0.01) (Fig. S4).

The median TTD of groups B and D was compared. Group A showed a significantly longer total TTD of 25.16 months than group D at 40.37 months (HR, 1.98; 95% CI, 1.74–2.25; p < 0.01) (Fig. 4c).

The median TTD of groups A and E was compared. There was no significant difference in the total TTD between group A at 25.16 months and group E at 21.29 months (HR, 0.91; 95% CI, 0.78–1.07; p = 0.26) (Fig. 4d).

## Discussion

In this study, we used a large-scale MDV database of 13,284 patients with HR-positive/HER2-negative ABC to evaluate the clinical significance of switching CDK4/6i + ET. Regarding the AEs of CDK4/6is, a large-scale study with more than 100,000 patients was conducted using VigiBase and the Food and Drug Administration Adverse Event Reporting System [20]. Regarding therapy patterns and treatment duration after switching to CDK4/6i + ET, a study was conducted using the MDV database; however, the sample size of 1,170 patients was insufficient [21]. Other studies on the effectiveness of switching to a CDK4/6i have also been conducted; however, none have involved more than 10,000 patients. To the best of our knowledge, this is the largest retrospective study that investigated the effectiveness of CDK4/6is, including therapy patterns and treatment duration. Blumenthal et al. demonstrated a correlation between TTD and survival endpoints, suggesting that TTD is an important indicator for evaluating therapeutic effectiveness and tolerability [22]. We found that switching to another CDK4/6i in the second-line setting after using CDK4/6i + ET in the first-line setting for HR-positive/HER2-negative ABC resulted in a significantly longer TTD than switching to ET monotherapy. Furthermore, the TTD did not change according to the order of palbociclib and abemaciclib administration in the first- and second-line settings.

Between groups A and B, switching from one CDK4/6i to another CDK4/6i (11.20 months) resulted in a significantly longer second-line TTD than switching to ET monotherapy (4.93 months). In the MAINTAIN trial, switching from palbociclib + ET (84%) or ribociclib + ET (11%) to ribociclib + FUL or EXE significantly prolonged the PFS in the second-line setting compared with switching to FUL or EXE monotherapy (5.3 vs. 2.8 months; HR, 0.57; 95% CI, 0.39–0.85; p < 0.01) [12]. In the post-MONARCH trial, switching from palbociclib + AI (59%) or ribociclib + AI (34%) to abemaciclib + FUL significantly prolonged the PFS in the second-line setting compared with switching to ET monotherapy (5.6 vs. 3.9 months; HR, 0.66; 95% CI, 0.48–0.91; p < 0.01) [13]. In the EMBER-3 trial, switching from palbociclib + AI (61%), ribociclib + AI (29%), or abemaciclib + AI (10%) to abemaciclib + imlunestrant significantly prolonged the PFS in the second-line setting compared with switching to imlunestrant monotherapy (9.1 vs. 3.7 months; HR, 0.51; 95% CI, 0.38–0.61) [14]. However, the EMBER-3 trial included patients who experienced disease progression while receiving AI (administered alone or with a CDK4/6i) as a neoadjuvant or adjuvant treatment within 12 months after completing adjuvant treatment or while receiving first-line treatment for ABC. We analyzed a larger, real-world cohort with more patients than those included in the clinical trials, and our findings were consistent with the results reported in these trials. A direct comparison was not possible; however, our TTD outcomes were superior to the PFS outcomes observed in these clinical trials. As clinical trials are strictly controlled by rigorous protocols, RECIST assessments are performed more frequently. By contrast, in real-world clinical practice, the frequency of RECIST assessments is relatively low and depends on the patient’s condition owing to factors such as the cost and radiation exposure. Consequently, the detection of disease progression (PD) may be delayed, which could be one of the reasons why our outcomes were superior. Additionally, the difference between the outcomes of this study and those of clinical trials is that group A showed a longer second-line TTD (11.20 months) compared with first-line TTD (8.06 months). At the Memorial Sloan Kettering Cancer Center in the United States, a retrospective comparative study was conducted on CDK4/6i switching, focusing on the TTD, to compare patients who discontinued first-line CDK4/6i therapy due to AEs with those who discontinued owing to PD. This study reported that the occurrence of AEs significantly shortened the TTD of first-line therapy. Furthermore, patients who discontinued first-line therapy due to AEs had a significantly longer TTD for second-line CDK4/6i therapy than those who discontinued treatment due to PD (patients who discontinued due to AEs: first-line TTD, 3.0 months; second-line TTD, 10.1 months and patients who discontinued due to PD: first-line TTD, 10.0 months; second-line TTD, 4.7 months). Based on their study, it is also likely that a certain number of patients in our study discontinued first-line CDK4/6i therapy due to AEs. This could be one of the reasons why the second-line TTD was longer than the first-line TTD in group A.

Next, between groups A and C, switching from one CDK4/6i to another CDK4/6i (11.20 months) resulted in a significantly longer second-line TTD than switching to CT (5.16 months). Some clinical trials have compared CDK4/6i therapy with CT. In the PEARL trial, palbociclib + ET failed to show PFS superiority over capecitabine in the second-line setting for patients with HR-positive/HER2-negative ABC (7.5 months vs. 10.0 months; HR, 1.13; 95% CI, 0.85–1.50) [23]. By contrast, in the Young-PEARL trial, palbociclib + EXE with ovarian function suppression for premenopausal patients with HR-positive/HER2-negative ABC significantly prolonged PFS compared with capecitabine therapy (19.5 months vs. 14.0 months; HR, 0.75; 95% CI, 0.57–0.98; p = 0.04) [24]. Similarly, in the RIGHT-Choice trial, ribociclib + ET with ovarian function suppression for premenopausal patients with HR-positive/HER2-negative ABC significantly prolonged PFS compared with CT (21.8 months vs. 12.8 months; HR, 0.61; 95% CI, 0.43–0.87; p = 0.03) [25]. The large cohort included in the present study also demonstrated the superiority of CDK4/6i therapy over CT, which is consistent with these findings. Therefore, switching to another CDK4/6i instead of CT in the second-line setting may remain a viable therapeutic option after first-line CDK4/6i therapy. The background characteristics of patients did not match in this study; therefore, further analyses using prospective data are required.

Between groups B and D, in the transition from first-line to second-line therapy, switching from ET to a CDK4/6i (40.37 months) resulted in a significantly longer total TTD of first- and second-line therapy than switching from a CDK4/6i to ET (20.50 months). A similar comparison was made in the SONIA trial, where the use of a CDK4/6i in the first-line setting did not show a significantly different PFS following the first- and second-line therapies compared with CDK4/6i therapy in the second-line setting for patients with HR-positive/HER2-negative ABC (31.0 months vs. 26.8 months; HR, 0.87; 95% CI, 0.74–1.03; p = 0.10) [26]. Rather, the median CDK4/6i therapy duration was 16.5 months longer in patients who received a CDK4/6i in the first-line setting (24.6 months) than in those who received a CDK4/6i in the second-line setting (8.1 months). Additionally, the first-line CDK4/6i group had grade 3 or higher AEs, and the per-patient drug costs were $200,000 higher. Based on these findings, the SONIA trial concluded that starting the therapy with ET monotherapy in the first-line setting instead of combining it with a CDK4/6i could effectively reduce drug toxicity and make the therapy more accessible to cost-restricted patients. In our study, the total TTD of first- and second-line therapies was longer in group D (the second-line CDK4/6i group), which differs from the findings of the SONIA trial. The primary reason group D showed a significantly longer TTD than group B can be attributed to differences in patient characteristics. Patients in group D may have had more favorable prognostic features, allowing for a prolonged response to first-line ET monotherapy, followed by extended treatment duration with CDK4/6i introduced in the second-line setting. In contrast, group B may have included patients with more advanced disease who required early initiation of CDK4/6i, potentially resulting in shorter treatment durations in both the first- and second-line settings.

Between groups A and E, in the transition from first-line to second-line therapy, there was no significant difference in the total TTD of first- and second-line therapies between switching from one CDK4/6i to another CDK4/6i (25.16 months) and switching from CT to a CDK4/6i (21.29 months). Patients in group E received CT as the initial therapy; therefore, it was likely that many patients with a visceral crisis were included. It remains unclear which group of patients should be selected for CDK4/6is instead of CT; however, this suggests that, even in cases of visceral crisis, some patients may be able to receive a CDK4/6i as first-line therapy instead of CT.

Developing resistance to hormone therapy is a major challenge in the treatment of HR-positive and HER2-negative ABC. The activation of the PI3K/AKT pathway has been recognized as a cause of hormone therapy resistance [27]. Several phase III trials (MONARCH-2, post-MONARCH, and EMBER-3) have investigated *PIK3CA/AKT1/PTEN*-mutated subgroups, and all have demonstrated the additional efficacy of abemaciclib, regardless of the *PIK3CA/AKT1/PTEN* mutations status [12–14]. Currently, targeted inhibitors of the PI3K/AKT pathway are available for HR-positive/HER2-negative ABC with *PIK3CA/AKT1/PTEN* mutations [16]. In the CAPItello-291 trial, capivasertib + FUL significantly prolonged PFS compared with FUL monotherapy in patients with HR-positive/HER2-negative ABC with *AKT1* mutations, but the clinical benefit remained insufficient (median PFS, 7.3 months vs. 3.1 months; HR, 0.60; 95% CI, 0.51–0.71; p < 0.001) [15]. In the SOLAR-1 trial, alpelisib + FUL significantly prolonged PFS compared with FUL monotherapy in patients with HR-positive/HER2-negative ABC with *PIK3CA* mutations (median PFS, 11.0 months vs. 5.7 months; HR, 0.65; 95% CI, 0.50–0.85; p < 0.001) [28]. Therefore, both agents have the potential to become key drugs for HR-positive/HER2-negative ABC. Although a direct comparison could not be made, our findings were comparable to those of CAPItello-291 and SOLAR-1 trials. Even in current real-world clinical practice, where treatment options have expanded based on the mutation status of *PIK3CA/AKT1/PTEN*, switching CDK4/6i remains an effective therapeutic option for HR-positive/HER2-negative ABC.

## Limitations

This retrospective study utilized real-world data, which presents several limitations. First, the MDV database does not allow for patient tracking across multiple institutions. Therefore, if a patient received treatment at more than one facility, there may be overlapping or missing treatment records. Additionally, as the reasons for treatment discontinuation were not recorded, we could not determine whether patients stopped therapy due to disease progression, adverse events, or other causes. Moreover, important clinical information essential for therapeutic decision-making, such as cancer stage, metastatic sites, number of metastases, and performance status, was not available. As a result, commonly used endpoints to assess therapeutic efficacy, including overall survival, progression-free survival, and tumor response, could not be evaluated. This is because the MDV database does not contain data on treatment outcomes. Given these limitations, the possibility of residual confounding from unmeasured factors cannot be excluded. We considered applying statistical adjustment methods such as propensity score matching or inverse probability weighting. However, the number of available covariates was limited, making it difficult to estimate propensity scores with sufficient precision. Furthermore, applying these methods without adequate covariate information may introduce additional bias rather than reduce it. Therefore, we decided not to apply these methods. Instead, we evaluated the robustness of our findings through subgroup analyses while accounting for potential confounding.

HR/HER2 status was not directly available in the claims database. Therefore, we defined HR-positive/HER2-negative breast cancer as patients who received ET but did not receive anti-HER2 therapies. Although this operational definition may not perfectly reflect biomarker status, it is consistent with previous database studies and considered a reasonable proxy within the constraints of the data source.

TTD is a useful surrogate endpoint that reflects both treatment effectiveness and tolerability in real-world data where information on disease progression or tumor response is unavailable [22], and thus its use in this study provides valuable insight. Previous studies have used TTD as a surrogate endpoint to assess the therapeutic effectiveness of CDK4/6is [29, 30]. However, TTD does not distinguish between the reasons for treatment discontinuation and may be influenced by physician or institutional practice patterns; therefore, its interpretation should be made with caution. Further prospective studies incorporating more detailed clinical and pathological data are warranted.

## Conclusion

This study demonstrates the therapeutic outcomes of CDK4/6i switching in patients with HR-positive/HER2-negative ABC in real-world clinical practice. Going forward, CDK4/6i switching will continue to be considered a viable therapeutic option for this patient population. These findings may contribute to future therapeutic strategies, including the optimization of therapy sequencing.

## Supplementary Information

Below is the link to the electronic supplementary material.

Supplementary file1 (DOCX 468 KB)

## Data Availability

The data that support the findings of this study are available from Medical Data Vision Co., Ltd. but restrictions apply to the availability of these data, which were used under license for the current study, and so are not publicly available. Data are however available from the authors upon reasonable request and with permission of Medical Data Vision Co., Ltd.
