# Supplementary material for: Clinical significance on switching CDK4/6 inhibitors among 13,284 patients with metastatic breast cancer
Source: Breast Cancer. 2025 Oct 12;32(6):1405–16. doi: 10.1007/s12282-025-01768-6 (PMC12552409; doi:10.1007/s12282-025-01768-6)
Supplement: Supplementary file 1 — Supplementary file1 (DOCX 468 KB) [file 12282_2025_1768_MOESM1_ESM.docx]

**Supplementary Table 1　Percentage of each regimen pattern**

**
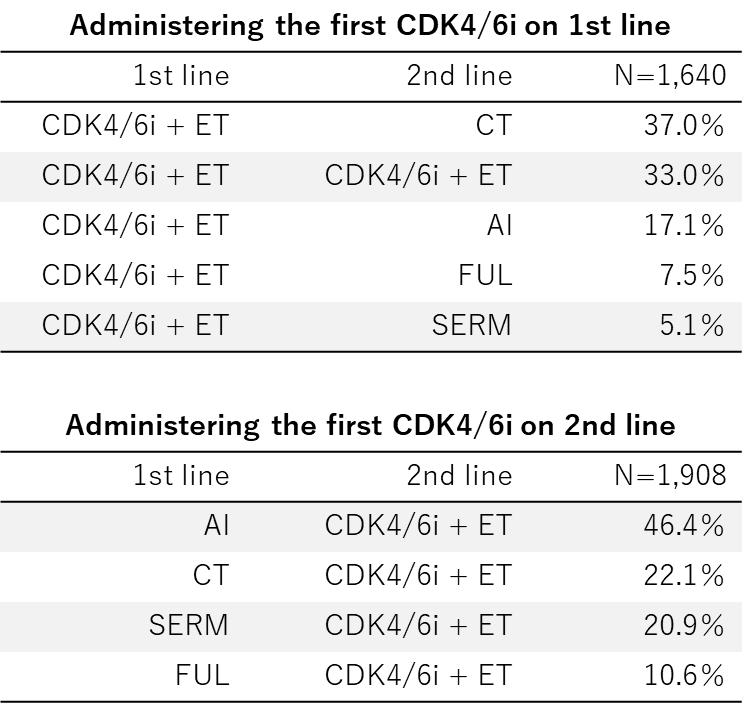
**

**Supplementary Figure 1　 Patterns of regimens in group A**


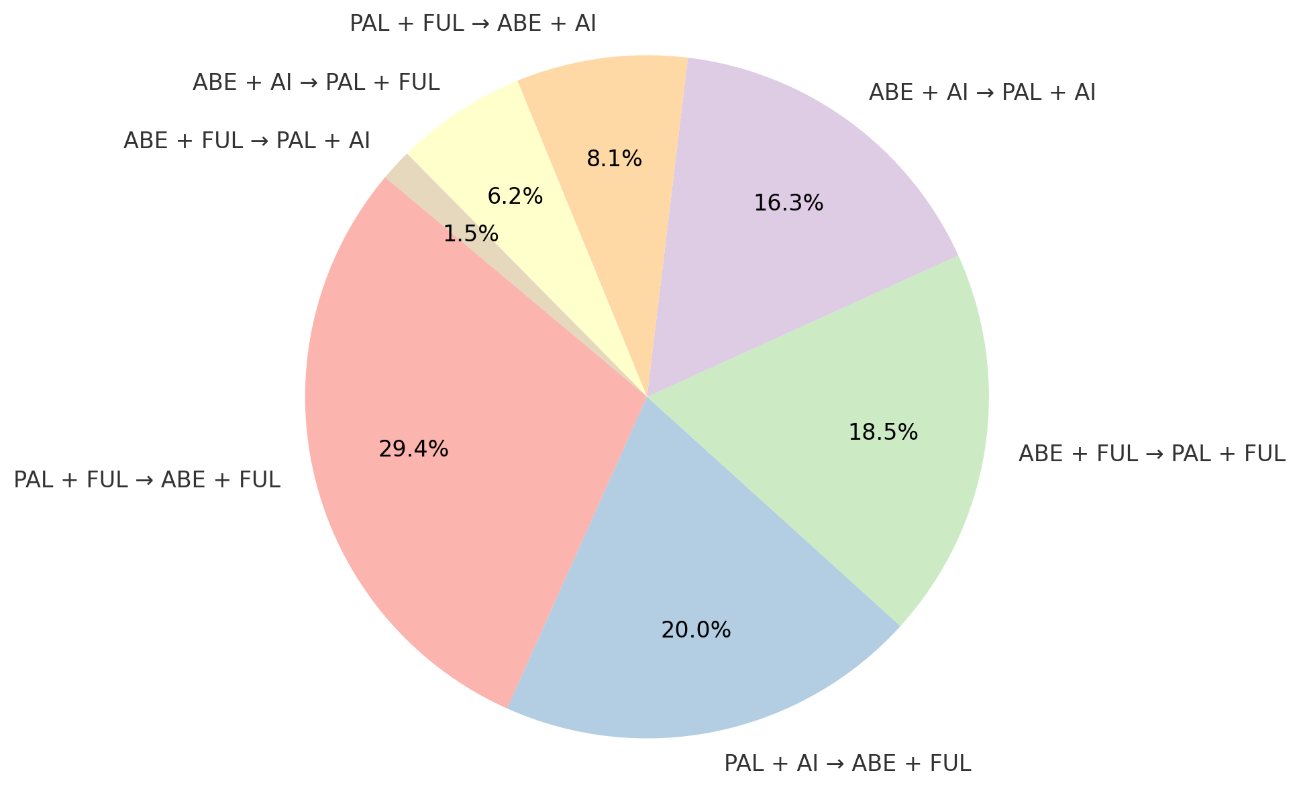


A pie chart illustrates the distribution of combined ET used with CDK4/6i and the sequencing of Abemaciclib and Palbociclib when switching CDK4/6i in group A patients. Combined ET is categorized as either Fulvestrant (FUL) or aromatase inhibitor (AI).

**Supplementary Figure 2　Total TTD of first- and second-line in group A and B**


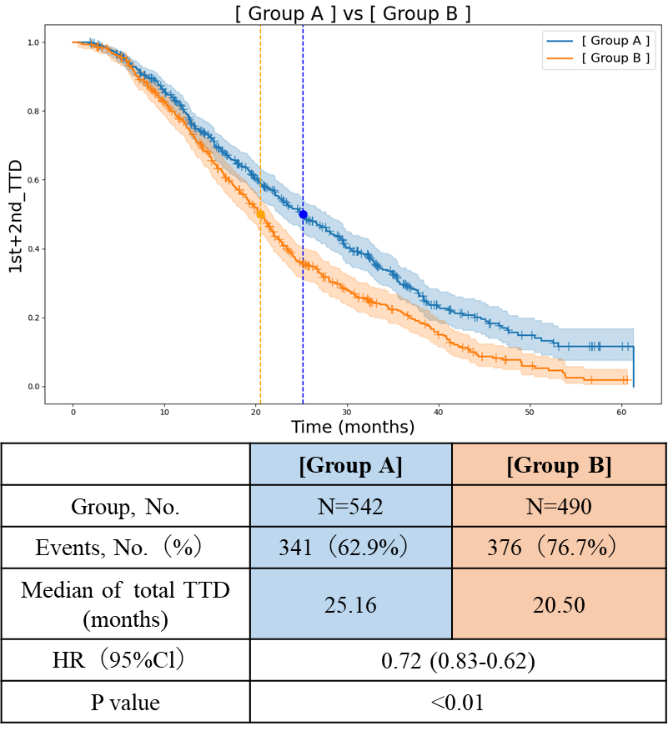


Kaplan-Meier estimates of total TTD of first- and second-line are shown for group A and B.

**Supplementary Figure 3a　 Second-line TTD on the order of palbociclib (PAL) and abemaciclib (ABE)**


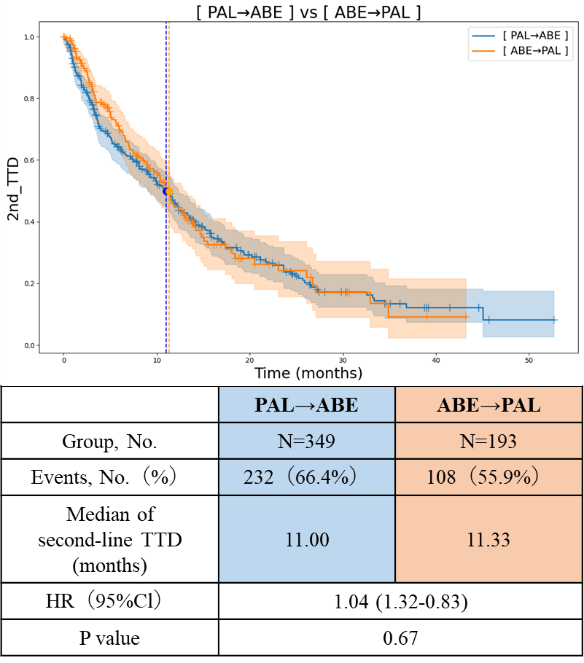


**Supplementary Figure 3b　 Total TTD of first- and second-line on the order of palbociclib (PAL) and abemaciclib (ABE)**


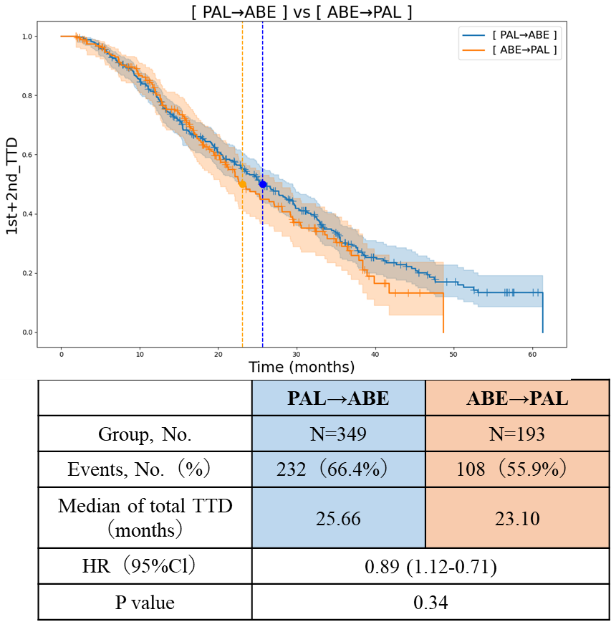


Kaplan-Meier estimates of TTD are shown for group A patients who switched from palbociclib to abemaciclib (PAL→ABE) and those who switched from abemaciclib to palbociclib (ABE→PAL). Supplementary Figure 3a shows second-line TTD, while Supplementary Figure 3b shows total TTD of first- and second-line.

**Supplementary Figure 4　Total TTD of first- and second-line in group A and C**


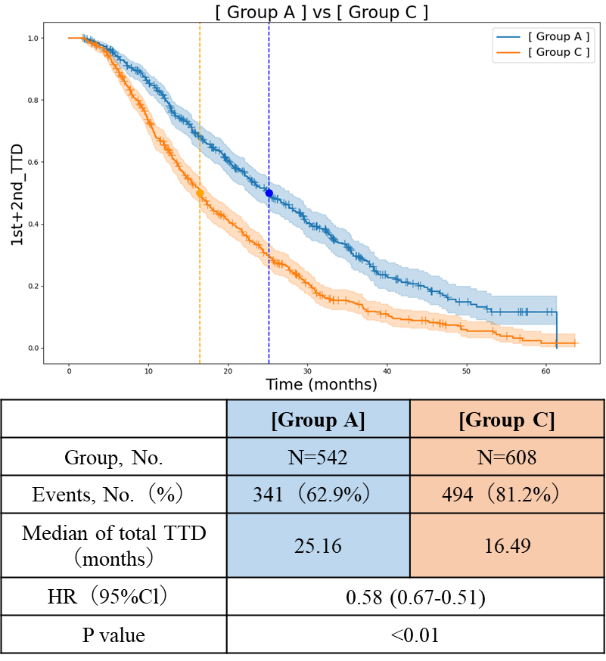


Kaplan-Meier estimates of total TTD of first- and second-line are shown for group A and C.
